# DNA Methyltransferases Regulate Pathogenicity of *Botrytis cinerea* to Horticultural Crops

**DOI:** 10.3390/jof7080659

**Published:** 2021-08-14

**Authors:** Zhanquan Zhang, Chang He, Yong Chen, Boqiang Li, Shiping Tian

**Affiliations:** 1 Key Laboratory of Plant Resources, Institute of Botany, Chinese Academy of Sciences, Beijing 100093, China; zhangzhanquan82@ibcas.ac.cn (Z.Z.); change@ibcas.ac.cn (C.H.); cy123898@163.com (Y.C.); bqli@ibcas.ac.cn (B.L.); 2 College of Life Sciences, University of Chinese Academy of Sciences, Beijing 100049, China

**Keywords:** *Botrytis cinerea*, DNA methyltransferases, synergistic effect, pathogenicity, development

## Abstract

*Botrytis cinerea* is one of the most destructive fungal pathogens that cause gray mold rot in horticultural products, including fresh fruits, vegetables, and flowers, leading to serious economic losses. *B. cinerea* is difficult to control because it has strong stress resistance and complex infection modes. The pathogenic mechanisms of *B. cinerea* have been revealed at multiple levels, but little is known at the epigenetic level. In this study, we first revealed the important role of DNA methyltransferases in regulating the development and pathogenicity of *B. cinerea*. We showed that two DNA methyltransferases, BcDIM2 and BcRID2, showed a strong synergistic effect in regulating the pathogenicity of *B. cinerea*. The double knockout mutant *ΔBcdim2rid2* showed slower mycelial growth, lower spore germination, attenuated oxidative tolerance, and complete pathogenicity loss on various hosts, which is related to the reduced expression of virulence-related genes in *ΔBcdim2rid2* and the induced resistance of the host. Although *B. cinerea* has multiple DNA methyltransferases, the global methylation level is very low, and few 5mC sites can be detected by BS-seq. These results first revealed the important role and the action mode of DNA methyltransferases in *B. cinerea*.

## 1. Introduction

Decay caused by pathogenic fungi is the main reason for the loss of horticultural products during the postharvest state. *Botrytis cinerea*, the causing agent of grey mold disease, is one of the most destructive fungal pathogens of important horticulture products, including fresh fruits, vegetables, and flowers, resulting in USD 10 billion to 100 billion in global losses each year [1,2]. The annual global cost of grey mold treatment easily exceeds EUR 1 billion [3]. Due to its scientific and economic importance, *B. cinerea* is regarded as the second most important fungal plant pathogen in a worldwide scientific survey and has been used as a model system to uncover the pathogenesis of necrotrophic pathogens [3]. During the past several decades, great efforts have been made to delve into the pathogenic mechanisms of *B. cinerea*. The pathogenicity of *B. cinerea* can be regulated by many factors, including signal transduction components [4,5], ROS generating systems [6,7,8,9,10], and protein secretion systems [11,12], but little is known about the regulation mechanisms at the epigenetic level.

Epigenetic modifications serve as a bridge between genetic components and the environment and are involved in many biological processes. Pathogen infection is one of the most complex and destructive stresses for plants. Epigenetic modifications are actively involved in the interactions of plant-pathogens. The epigenetic marks, such as histone acetylation, histone methylation, and DNA methylation, play crucial roles in plant immunity against pathogens [13,14,15]. In the model fungi *Saccharomyces cerevisiae*, *Schizosaccharomyces pombe,* and *Neurospora crassa*, the epigenetic regulation has been extensively studied [16,17,18]. From the perspective of pathogens, epigenetics is distinctly at the forefront of human pathogens. By comparison, the understanding of the function of epigenetics in modulating the pathogenicity of plants is still in its infancy [19]. However, some available research suggests that epigenetic factors also play critical roles in regulating the pathogenicity of plants. *Phytophthora ramorum*, a plant pathogen causing forest disease, showed a significant difference in virulence among different isolates, although they had an extreme genetic similarity, even within the same genotype [20], which suggests that the pathogenicity could be regulated at the epigenetic level. There are many examples showing that the avirulence genes of plant pathogens gain virulence without sequence changes occurring within the open reading frame [21,22,23,24,25,26]. Epigenetic regulation of expression allows the pathogen to successfully cope with the new immune capabilities of the host while retaining the avirulence genes. These results suggest that epigenetic systems offer a versatile means for reversibly regulating the expression of effector genes of plant pathogens in response to environmental change.

DNA methylation is an important epigenetic modification involved in many biological characters, including genomic imprinting, X-chromosome inactivation, silencing of transposons, gene regulation, and development [27,28,29,30]. In many fungi, including *Beauveria bassiana*, *Cryphonectria parasitica*, *Mucor Rouxii*, *Yarrowia* *lipolytica*, and *Ustilago maydis*, the DNA methylation patterns were closely related with the developmental stages [31,32]. In *N. crassa* and *Magnaporthe oryzae*, 1.5% and 0.22% cytosines in genomes were methylated, respectively, and the 5mCs were associated with gene expression, transposon silencing, and heterochromatin formation [16,33]. In eukaryotic organisms, DNA methylation almost exclusively occurs at position 5 of the cytosine base (C5) and is usually deposited in CG, CHG, and CHH contexts [34,35]. Cytosine’s methylation, as an evolutionarily conserved epigenetic modification in biological kingdoms, appears to be obligatory in plants and shows comparatively higher methylation levels [36]. DNA methylation usually occurs within the promoter region or gene-body in higher eukaryotes; it functions in regulating gene expression and silencing transposons and repeated elements. Silencing transposon appears to be the main purpose of DNA methylation in fungi. Unusual deposition patterns occur in the dimorphic yeast *Candida albicans*, where DNA methylation has been shown to target and modulate the transcription of genes [37].

DNA methyltransferases (MTases) transfer methyl groups from *S*-adenosyl methionine to the 5-position of cytosine. The DNA MTase DNMT1 participates in the maintenance of existing genomic methylation, while DNMT3 is involved in de novo DNA methylation [38]. In *Arabidopsis* spp., the DNMT1 homolog MET1 regulates the seed development process [39]. In fungi, the DNMT1 family of MTases has been identified, but the DNMT3 family protein has not been found. DIM-2 (defective in methylation-2) and RID (RIP defective) are two DNA methyltransferases derived from fungi and show very high conservation in ascomycetes. In *N. crassa*, a model system for the investigation of DNA methylation, DIM-2 was closely associated with all DNA methylations in vegetative tissues [40], and RID was required for RIP (repeat-induced point mutation) during the sexual phase [41]. In another model fungus, *M. oryzae*, DIM-2 was responsible for most of the cytosine methylation, and RID was involved in the methylation of a small number of the cytosine sites. Interestingly, deletion of RID in *M. oryzae* changed the position of about 25% of the methylation sites that were present in the wild-type strain, implying there was an interaction or cooperation between these two methyltransferases [33]. Analogously, in the entomopathogenic fungus *Metarhizium robertsii*, RID regulated the specificity of DNA methylation, DIM-2 was responsible for most DNA methylation, and the double mutant of DIM-2 and RID showed an additive effect [42]. These results suggested that DIM-2 and RID had close cooperation in regulating the DNA methylation of fungi. The homologous proteins of DIM-2 and RID have also been identified in *Aspergillus nidulans*, *Cryphonectria parasitica,* and *Cordyceps militari*, and they played important roles in sexual development, secondary metabolites, and pathogenicity [43,44,45]. The biological functions of DNA MTases in filamentous fungi are diversified. In *Neurospora*, though the DNA MTase DIM-2 was responsible for all known DNA methylation, mutations of *dim-2* did not cause a detectable phenotype [40]. Curiously, the deletion of MTase genes in some fungi with low methylation levels resulted in obvious phenotypic changes [33,46]. These results indicated that the methylation level and function of methyltransferases in fungi were not conserved.

In order to fill the knowledge gap of epigenetic regulation in *B. cinerea*, we have deciphered the biological functions of DNA MTases and the methylation pattern of *B. cinerea* in this study. We investigated the functions of 5mC MTase genes of *B. cinerea* involved in regulating development and pathogenicity and unraveled the unknown mode of action of DNA MTases in this important pathogen.

## 2. Materials and Methods

### 2.1. Strains and Culture Conditions

*B. cinerea* strain B05.10 was used as the recipient strain for gene replacement and wild-type in this study. The wild-type and mutant strains of *B. cinerea* were generally maintained on potato dextrose agar (PDA) plates at 22 °C. Potato dextrose broth (PDB) was used to culture the conidia for germination assay. Conidia were collected in sterile distilled water and cleared from mycelium by filtration through two layers of sterile gauze. The concentration of conidia was determined with a hemacytometer.

### 2.2. Phylogenetic Analysis

The alignment of DNA MTase sequences was conducted using ClustalX 2.1. The phylogenetic tree was generated by MEGA 4.0, and bootstrap analysis was carried out by 1000 replications.

### 2.3. 5-Azacytidine Treatment

Conidia of the wild-type were cultured in PDB medium containing 0, 0.5 mm, 1 mm, and 2 mm 5-azacytidine at 22 °C for 2 to 8 h at 160 rpm. The treatment of cytidine was used as a negative control. The conidia were then collected and washed twice with sterile distilled water before detecting the germination rate and pathogenicity.

### 2.4. Construction of Knockout Mutants

Knockout mutants were obtained using a gene replacement strategy as described previously [47]. Single knockout mutants of DNA MTase genes were first generated using hygromycin B as the selection marker (Figure S1A). To get double mutants, the second target genes were knocked out with nourseothricin as the selection marker (Figure S1B). The primers used for the generation of knockout mutants are listed in Supplementary Table S1. Flank-spanning PCR was used to verify the correct insertion in mutants using the primer pairs that were located outside the L flank and inside the resistance cassette, respectively (Table S1). In order to exclude ectopic integration, the homokaryotic transformants were subjected to Southern blot analysis according to the method described previously [11].

### 2.5. Expression Analysis

Total RNAs were extracted using TRIzol Reagent (Tiangen Biotech, Beijing, China). For detecting the expression of virulence-related genes and resistance genes, pre-wounded tomato fruits were inoculated with 10 μL conidial suspension at a concentration of 10^7^ spores/mL. After inoculation (0, 8, 12, and 24 h), the pulps within 5 mm (diameter) around the infection sites were cut, and the total RNAs were extracted. Then, a PrimeScript^TM^ RT reagent Kit with gDNA Eraser (Takara, Tokyo, Japan) was used to synthesize first-strand cDNA. Quantitative PCR was conducted in a 20-μL reaction volume with SYBR Premix Ex Taq (Takara, Tokyo, Japan). The reaction was performed on the Applied Biosystems 7500 Real Time PCR System (Applied Biosystems, Foster City, CA, USA). Specific primers (Table S2) were designed using Primer Express software 3.0. The following PCR conditions were applied: 95 °C/10 min, 40 cycles of 95 °C/15 s, and 60 °C/30 s. Tubulin/actin genes of *B. cinerea* and actin gene of tomato were used as endogenous controls. The relative expression levels of target genes were calculated by the 2^−ΔΔC^^T^ method [48]. Each experiment included three biological repeats.

### 2.6. Virulence Assay

The virulence of wild-type and mutant strains was assayed on apple fruit (*Malus pumila* Mill cv. Fuji), tomato fruit (*Lycopersicon esculentum* Mill cv Castlemart), tomato leaves, and strawberry leaves. Before inoculation, *B. cinerea* strains were cultured for 10 days on PDA at 22 °C. Conidia were harvested with PDB media and adjusted in suspension to 5 × 10^3^ spores/mL. A 10 μL droplet of conidial suspension was inoculated in the pre-wounded apple or tomato fruit and incubated at 25 °C in enclosed plastic trays in order to maintain high relative humidity (95%). Detached leaves of 4-week-old tomato plants were inoculated with 5 μL of conidial suspensions. The leaves were incubated in petri dishes covered with soaked filter paper at 25 °C. Disease symptoms were scored each day. Each treatment contained three replicates with 5 fruits or leaves per replicate. For onion epidermis penetration assays, tiny pieces of onion epidermises were sliced and placed on glass slides, keeping the inner side face-up. Conidial suspensions were inoculated on the onion epidermis. The inoculated onion epidermises were incubated on humidified plates for 16 h at 25 °C. Epidermises were then dyed with Cotton Blue for 5 min. The penetrations were observed under a microscope after removing spare dye by washing with distilled water.

### 2.7. ROS Detection

The ROS of leaves were detected by diaminobenzidine (DAB) staining. The detached tomato leaves were inoculated with 5 μL conidial suspensions at a concentration of 5 × 10^3^ spores/mL. Subsequently, hydrogen peroxide was detected at 24 and 36 h after inoculation by DAB staining [49]. Infected tomato leaves were soaked in 1 mg/mL DAB solution overnight. The leaves were then immersed in ethanol to remove chlorophyll until they were suitable to image.

### 2.8. Quantification of Global DNA Methylation

Global DNA methylation was quantified using the MethylFlash^TM^ Methylated DNA Quantification Kit (Epigentek, New York, NY, USA). Total DNA was extracted using a DNeasy Plant Mini Kit (QIAGEN, Hilden, Germany). Briefly, 100 ng DNA was added and fixed on a strip well with a specific affinity for DNA. The DNA methylation level was then quantified by a 5mC capture antibody and a detection antibody. The amount of methylated DNA is proportional to the optical density (OD) values obtained from the enzyme-linked immunosorbent assays and is presented according to the calculated percentage of 5mC.

### 2.9. Whole-Genome Bisulfite Sequencing

Five micrograms of genomic DNA, extracted from 48-h-old mycelium, were used for high-throughput bisulfite sequencing in each sample. For library construction, the genomic DNA was fragmented by sonication using a Bioruptor (Diagenode, Liege, Belgium) to a mean size of approximately 250 bp, followed by blunt-ending, dA addition to the 3′-end, and adaptor ligation. Bisulfite conversion was carried out with an EZ DNA Methylation-Cold kit (Zymo Research), lambda DNA was used as control. The library was sequenced using Illumina HiSeq 4000 Genome Analyzer after desalting, size selecting, PCR amplification, and a second size selection. Raw sequencing data were processed by the Illumina base-calling pipeline. The clean data were mapped to the reference genome by BSMAP, and duplication reads were removed before merging the mapping results according to each library. Methylation levels were determined by dividing the number of reads covering each mC by the total reads covering that cytosine.

### 2.10. Statistical Analysis of Data

Data were analyzed using SPSS version 11.5 (SPSS Inc., Chicago, IL, USA). One-way analysis was conducted to determine the significance of the difference. Mean separation was performed by Duncan’s multiple range tests. The difference was considered significant when *p* < 0.05.

## 3. Results

### 3.1. DNA Methylation Is Involved in the Infection Process of B. cinerea

**Figure 1:**
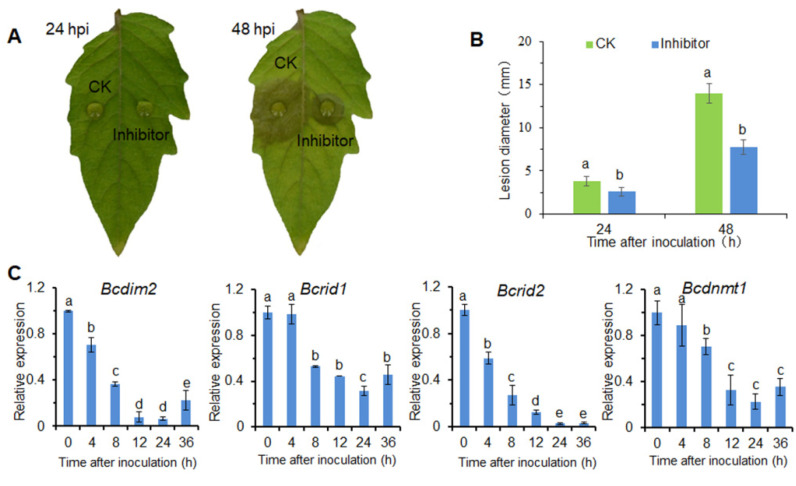
DNA methylation is involved in the infection process of *B. cinerea*. (**A**) Disease symptoms of detached tomato leaves after inoculation with 5-Aza-treated (1 mm) and non-treated conidia. (**B**) Statistical analysis of lesion diameter caused by 5-Aza-treated (1 mm) and non-treated conidia on detached tomato leaves. (**C**) Relative expression of DNA methyltransferase genes during infection processes of *B. cinerea*. Vertical bars represent standard errors of the means. Columns with different letters indicate significant differences (*p* < 0.05).

In order to determine whether DNA methylation was involved in the infection process of *B. cinerea*, we treated conidia with 5-azacytidine (5-Aza), a DNA methylation inhibitor, and then measured the virulence on detected tomato leaves. The results showed that DNA methylation inhibitor 5-Aza significantly decreased the virulence of *B. cinerea* on tomato leaf (Figure 1A). After 48 h of inoculation, the lesion diameter of the inhibitor-treated group was decreased by 45% compared to the CK group (Figure 1B).

By searching the genome database of *B. cinerea*, we found that four proteins Bcin15p00450, Bcin03p04600, Bcin09p05050, and Bcin09p01910, contain the conserved domain of C-5 cytosine methyltransferase (Figure S2A). The phylogram showed that these DNA MTases were grouped into several distinct clades (Figure S2B). According to the evolutionary relationship of these proteins and existing reports [50], these MTases were named BcDIM2 (Bcin15p00450), BcRID1 (Bcin03p04600), BcRID2 (Bcin09p05050), BcDNMT1 (Bcin09p01910), respectively. These results indicated that *B. cinerea* had more 5mC MTases than other known fungi, and two RIDs were first found in fungi [50]. During the infection process, the expressions of the four DNA MTase genes were gradually down-regulated (Figure 1C). The expression pattern of the MTase genes during the infection process is the result of the interaction between pathogen and host. These results suggested that DNA methylation was involved in the pathogenesis of *B. cinerea*.

### 3.2. DNA Methylation Is Involved in the Development of B. cinerea

**Figure 2:**
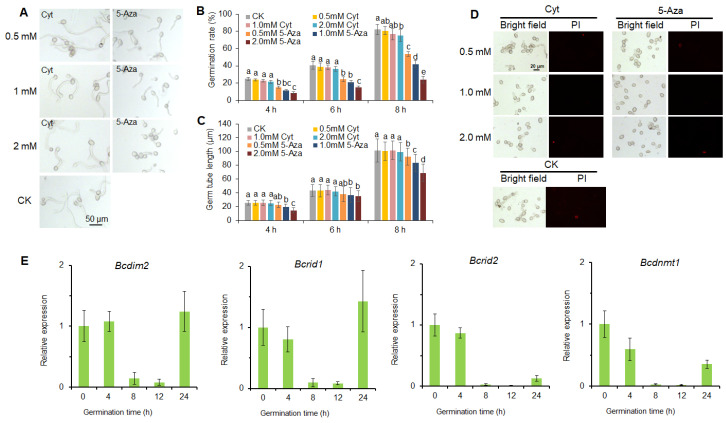
DNA methylation is involved in the development of *B. cinerea*. (**A**) The effect of 5-azacytidine on conidial germination of *B. cinerea*. The conidia of *B. cinerea* were incubated in potato dextrose broth (PDB) medium containing 0.5, 1, and 2 mm 5-azacytidine (5-Aza). Cytidine-treated (Cyt) conidia were used as negative control, and the untreated conidia were used as blank control (CK). (**B**) Conidial germination rate of *B. cinerea* after treating with 5-Aza and Cyt. (**C**) Germ tube length of *B. cinerea* after treating with 5-Aza and Cyt. (**D**) Cytotoxicity test of 5-azacytidine on *B. cinerea*. The conidia were stained with propidium iodide (PI) after treating with 5-azacytinde (5-Aza). Cytidine (Cyt) was used as negative control, and the untreated conidia were used as CK. (**E**) Relative expression of DNA methyltransferase genes during conidial germination of *B. cinerea*. Vertical bars represent standard errors of the means. Columns with different letters indicate significant differences (*p* < 0.05).

DNA methylation inhibitor 5-Aza could inhibit conidial germination, while the negative control cytidine (an analog of 5-Aza that does not inhibit DNA methylation) had no effect on the germination process (Figure 2A–C). Meanwhile, we tested whether 5-Aza and cytidine were cytotoxic to *B. cinerea* (Figure 2D). The results indicated that the concentrations of 5-Aza and cytidine used in this study had no cytotoxicity to *B. cinerea* (Figure 2D). The expression of the DNA MTase gene was relatively stable at the early stage of germination (0–4 h) and notably down-regulated within 8–12 h during the germination process (Figure 2E). Afterward, the expression of these genes increased rapidly after the germinated conidia were transferred to vegetative growth (Figure 2E). These results suggested that DNA methylation was involved in the germination of conidia, and DNA MTases played a negative role in this process.

### 3.3. Virulence Assay of DNA MTase Mutants on Fruit Hosts

**Figure 3:**
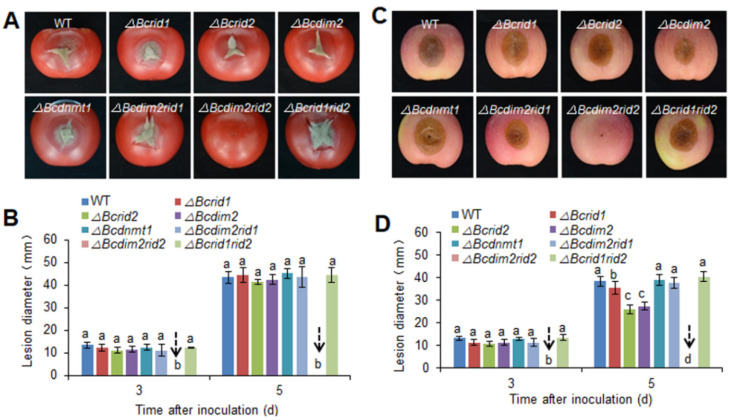
Virulence assay of DNA MTase mutants on fruit hosts. (**A**) Disease symptoms on tomato fruits 5 d after inoculation. (**B**) Lesion diameters on tomato fruits. (**C**) Disease symptoms on apple fruits 5 d after inoculation. (**D**) Lesion diameters on apple fruits. Vertical bars represent standard errors of the means. Columns with different letters indicate significant differences (*p* < 0.05).

To explore the biological function of DNA MTase genes in *B. cinerea*, we first constructed the single knockout mutants of four DNA MTase genes. The insertion sites in mutants were verified by flank-spanning PCR diagnosis (Figure S3). Southern blot analysis indicated that there were no ectopic integrations in the mutants (Figure S4). The virulence of mutants was tested on tomato and apple fruits. Single deletions of DNA MTase genes had no significant effect on the virulence of *B. cinerea* on tomato fruit, the virulence of *ΔBcdim2* and *ΔBcrid2* was slightly reduced on apple fruit (Figure 3). DIM2 and RID are two conserved DNA MTases in fungi, and previous reports have implied that synergistic effects may exist between them [33,42]. Therefore, in order to further explore the biological function of MTases in *B. cinerea*, we further constructed three double knockout mutants, *ΔBcdim2rid1,* *ΔBcrid1rid2,* and *ΔBcdim2rid2*. Interestingly, *Bcdim2* and *Bcrid2* showed a strong concerted action in regulating pathogenicity. The double knockout mutant *ΔBcdim2rid2* completely lost virulence on tomato and apple fruits, whereas the other two double knockout mutants showed comparable virulence to wild-type (Figure 3). No obvious lesion diameters were detected in these fruits inoculated by *ΔBcdim2rid2* even at 5 dpi (Figure 3).

### 3.4. Verification of the Synergistic Effect of Bcdim2 and Bcrid2 on Leaf Hosts

**Figure 4:**
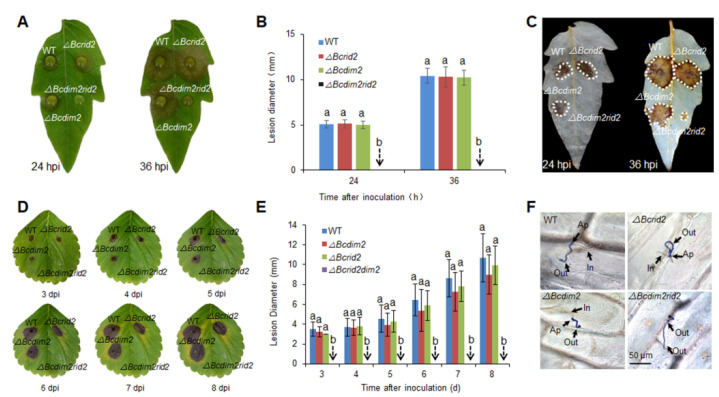
Verification of the synergistic effect of *Bcdim2* and *Bcrid2* on leaf hosts. (**A**) Disease symptoms on detached tomato leaves. (**B**) Lesion diameters on detached tomato leaves. (**C**) Detection of ROS accumulation around the infectious sites of tomato leaves after inoculation. The infected tomato leaves were stained by DAB after 24 and 36 h of inoculation. (**D**) Disease symptoms on detached strawberry leaves. (**E**) Lesion diameters on detached strawberry leaves. (**F**) Onion epidermis penetration assay. The onion epidermises were stained by cotton blue after 16 h of inoculation. In: the hyphae infected into the onion epidermis cells (brown color); Out: the hyphae outside the onion epidermis cells (blue color); Ap: appressoria. Vertical bars represent standard errors of the means. Columns with different letters indicate significant differences (*p* < 0.05).

To further verify the synergistic effect of *Bcdim2* and *Bcrid2* in regulating the pathogenicity of *B. cinerea*, the virulence of *ΔBcdim2*, *ΔBcrid2,* and *ΔBcdim2rid2* was detected on detached tomato and strawberry leaves. Similar to the assays on fruit hosts, single deletions of *Bcdim2* or *Bcrid2* had no effect to the virulence of *B. cinerea*, and double knockout of *Bcdim2* or *Bcrid2* resulted in complete non-pathogenicity on leaf hosts (Figure 4A,B,D,E). *ΔBcdim2rid2* could not cause visible disease lesions on strawberry leaves even at 8 dpi (Figure 4D). Reactive oxygen species (ROS) production around infectious sites on tomato leaves were detected (Figure 4C). The results indicated a large amount of ROS accumulation around the infectious sites inoculated by the wild-type strain and the single knockout mutants, which is beneficial to the colonization of *B. cinerea*. In comparison, no ROS was detected in the site inoculated with *ΔBcdim2rid2* at 24 h; only a little ROS was tested at 36 h (Figure 4C). The results of the onion epidermis penetration assay indicated that single deletion of *Bcdim2* or *Bcrid2* did not affect the penetration process, whereas the double deletion of *Bcdim2* and *Bcrid2* resulted in the inability of penetration (Figure 4F). These results indicated that there was a strong concerted action between *Bcdim2* and *Bcrid2* in the interaction between *B. cinerea* and hosts.

### 3.5. DNA MTases Ais Attenuatedre Involved in the Development of B. cinerea

**Figure 5:**
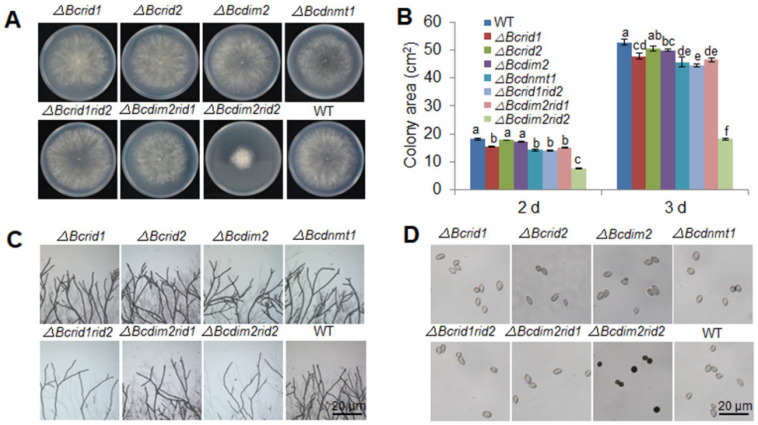

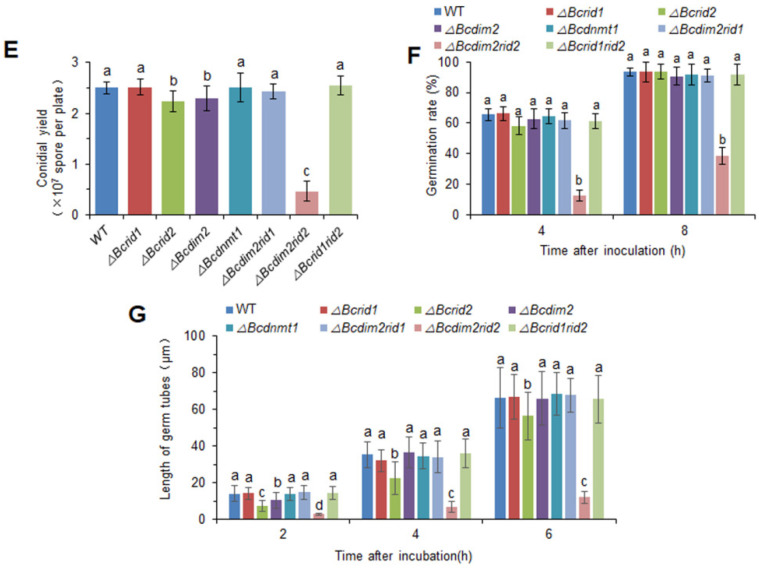
DNA MTases affect the vegetative growth and conidiation of *B. cinerea*. (**A**) Colony morphology of wild-type and mutants after culturing for 4 d on PDA plates. (**B**) Statistical analysis of radial growth of wild-type and mutants. (**C**) Mycelial morphology of wild-type and mutants. (**D**) Conidial morphology of wild-type and mutants. (**E**) Conidial yield of wild-type and mutants after culturing for 14 d. (**F**) Statistical analysis of the germination rates of wild-type and mutants. Vertical bars represent standard errors of the means. (**G**) The tube length of different mutants at 2 h, 4 h, and 6 h after inoculation. Columns with different letters indicate significant differences (*p* < 0.05).

The vegetative growths of four single knockout mutants were not obviously affected (Figure 5A,B). The growth rate of *ΔBcdim2rid2* was severely inhibited (Figure 5A,B), and *ΔBcdim2rid2* showed more sparse hyphae density at the colony edge on PDA plates (Figure 5C). The color of *ΔBcdim2rid2* conidia was black under microscopic examination (Figure 5D). The conidiation of *ΔBcdim2rid2* was significantly decreased (Figure 5E). In addition, the germination rate and tube length of *ΔBcdim2rid2* spores showed to be significantly lower as compared to that of wild-type (Figure 5F,G). These results suggested that *Bcdim2* and *Bcrid2* were involved in the development of *B. cinerea*, *Bcdim2,* and *Bcrid2* act in concert with each other.

### 3.6. The Oxidative Tolerance of ΔBcdim2rid2 Is Attenuated

**Figure 6:**
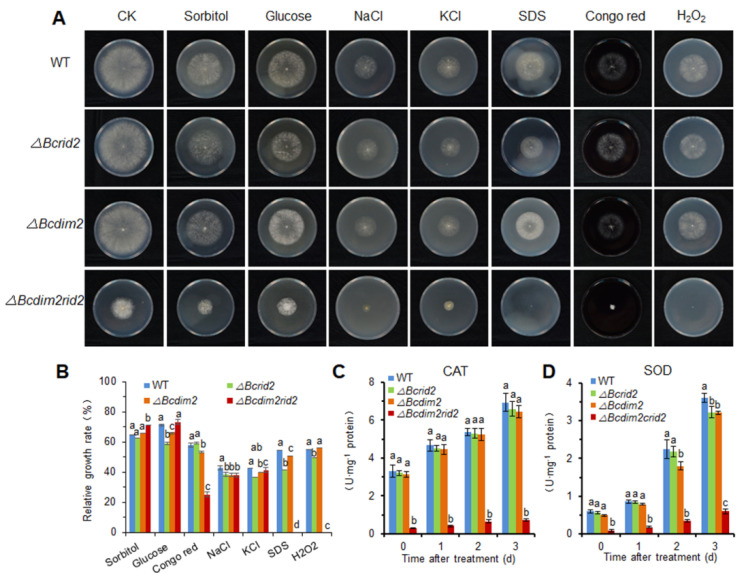
The oxidative tolerance of *ΔBcdim2rid2* is significantly decreased. (**A**) Tolerance test of wild-type and mutants to osmotic stress (1 M sorbitol, 1 M glucose, 1 M NaCl, and 1 M KCl), cell wall stress (2 mg/mL Congo red, 0.02% SDS), and oxidative stress (10 mm H_2_O_2_). (**B**) Relative growth rates of wild-type and mutants under different stressors. (**C**) The activities of catalase (CAT) in wild-type and mutants. (**D**) The activities of superoxide dismutase (SOD) in wild-type and mutants. Vertical bars represent standard errors of the means. Columns with different letters indicate significant differences (*p* < 0.05).

To analyze the effect of DNA MTases on stress tolerance of *B. cinerea*, wild-type and mutant strains were cultured on PDA plates under different stress conditions, including oxidative stress (10 mm H_2_O_2_), osmotic stress (1 M sorbitol, 1 M glucose, 1 M NaCl, 1 M KCl), and cell wall stress (2 mg/mL Congo red, 0.02% sodium dodecyl sulfate (SDS)) (Figure 6A). Stress tolerance of *ΔBcdim2rid2* was significantly decreased to some stressors, including Congo red, SDS, and H_2_O_2_. The relative growth rate of the *ΔBcdim2rid2* was decreased by 56% compared with the wild-type strain under Congo red stress. Under stresses of SDS and H_2_O_2_, the growth of *ΔBcdim2rid2* was completely inhibited (Figure 6B). The activities of ROS scavengers, catalase (CAT), and superoxide dismutase (SOD) were not affected in single mutants compared to the wild-type and were severely suppressed in *ΔBcdim2rid2*, which led to the reduced tolerance to oxidative stress (Figure 6C,D).

### 3.7. Expression of Pathogenic Genes in ΔBcdim2rid2 Is Inhibited

**Figure 7:**
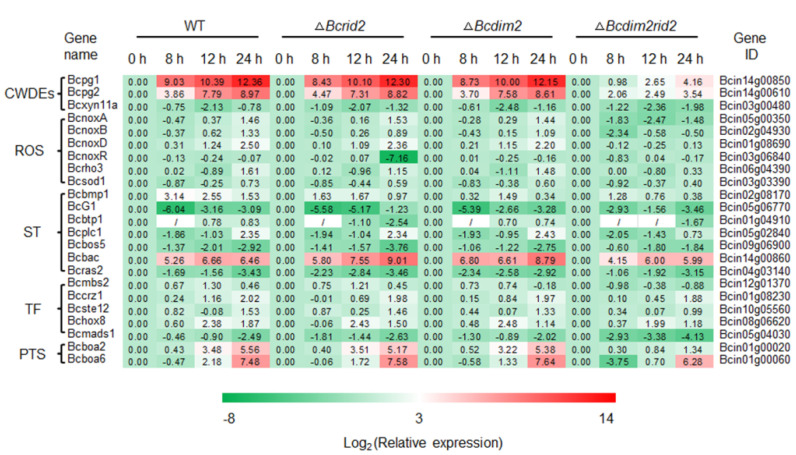
The pathogenic genes are suppressed during the interaction between *ΔBcdim2rid2* and host. CWDE: cell wall degrading enzyme gene; ROS: ROS metabolism related gene; ST: signal transduction component; TF: transcriptional factor; PTS: phytotoxin synthesis gene.

To further unravel the underlying mechanisms of DNA MTases regulating the pathogenicity of *B. cinerea*, we examined the expression of a set of virulence-related genes during the interaction between mutants and tomato fruit. A total of 23 genes were detected, including cell wall-degrading enzyme genes (CWDEs), ROS metabolism-related genes (ROS), signal transduction components (ST), transcriptional factors (TF), and phytotoxin synthesis genes (PTS). The results indicate that the expression of most of these virulence-related genes was up-regulated during the infection process of the wild-type strain and single knockout mutants (*ΔBcdim2* and *ΔBcrid2*) (Figure 7). In particular, the expression of endopolygalacturonase genes (*Bcpg1* and *Bcpg2*), botcinic acid synthesis genes (*Bcboa2* and *Bcboa6*), and NADPH oxidase complex subunit genes (*BcnoxA*, *BcnoxB,* and *BcnoxD*) increased significantly in the wild-type strain, *ΔBcdim2,* and *ΔBcrid2* (Figure 7). By contrast, the expression of these virulence-related genes was sharply suppressed in *ΔBcdim2rid2*. The expression of *Bcpg1* and *Bcpg2* in the wild-type strain increased by 5274-fold and 503-fold, respectively, at 24 h after inoculation compared with the initial level. In comparison, the expression of *Bcpg1* and *Bcpg2* in *ΔBcdim2rid2* only increased by 18-fold and 12-fold, respectively, at the same time point (Figure 7). Relative to wild-type, the expression of NADPH oxidase genes (*BcnoxA*, *BcnoxB,* and *BcnoxD*) also showed a decreasing trend during the infection process in *ΔBcdim2rid2* (Figure 7). Furthermore, the activities of some extracellular pathogenic proteins (PG, PME, and Cx) were also significantly reduced in the double knockout mutant (Figure S5). In the single mutants *ΔBcdim2* and *ΔBcrid2*, the expression patterns of these virulence genes were similar to that in the wild-type strain. These results suggested that *Bcdim2*, *Bcrid2* were involved in the regulation of the expression of pathogenic genes in *B. cinerea*, and there was strong cooperation between *Bcdim2* and *Bcrid2*.

### 3.8. ΔBcdim2rid2 Induces the Resistant of Host

**Figure 8:**
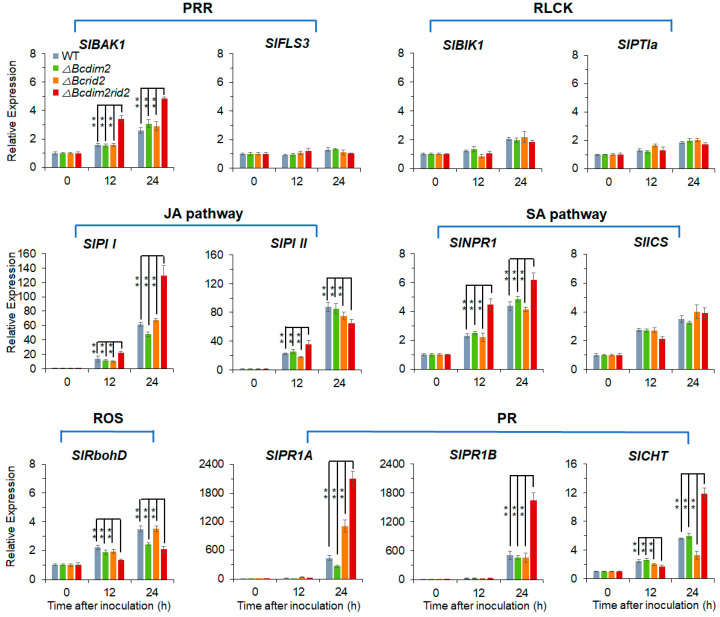
The expression of resistant genes in fruit is induced by *ΔBcdim2rid2*. PRR: pattern recognition receptors (or coreceptor); RLCK: receptor-like cytoplasmic kinases; JA pathway: jasmonic acid pathway components; SA pathway: salicylic acid pathway components; ROS: NADPH oxidase; PR: pathogenesis-related genes. Asterisks indicate significant differences between *ΔBcdim2rid2* and other strains (** *p* < 0.01).

Furthermore, we tested the immune response of fruit during the interaction with different strains. We examined the expression patterns of several components in resistant systems, including pattern recognition receptors (PRR), receptor-like cytoplasmic kinases (RLCK), jasmonic acid pathway components (JA), salicylic acid pathway components (SA), NADPH oxidase (ROS), and pathogenesis-related genes (PR). PRRs are cell-surface immune receptors, which are responsible for the perception of microbe- or host-derived immunogenic molecular patterns. WT and single knock mutants induced the expression of PRR coreceptor *SlBAK1* in fruit, while *ΔBcdim2rid2* significantly enhanced this induction effect (Figure 8). All strains weakly induced RLCKs, and there was no significant difference between *ΔBcdim2rid2* and other strains (Figure 7). Necrotrophic pathogens mainly activated the JA/ethylene resistance signaling pathway. The results suggest that *B. cinerea* stimulated the marker genes *SlPI I* and *SlPI II* of the JA pathway. Double knockout of *Bcdim2*/*Bcrid2* increased the induction effect on *SlPI I* (Figure 8). Interestingly, we also found that all strains could induce the marker gene *SlNPR1* of the SA pathway and the SA biosynthesis-related gene *SlICS*. Similarly, *ΔBcdim2rid2* promoted the expression of *SlNPR1* compared with wild-type and single knockout mutants (Figure 8). NADPH oxidase RbohD is responsible for the ROS burst of plants attacked by pathogens. In the fruit infected by *ΔBcdim2rid2*, the expression of *SlRbohD* was significantly lower than that in WT and single knockout mutants (Figure 8). SlPR1A/B and SlCHT (chitinase) are pathogenesis-related proteins of plants and contribute to the plants’ resistance. *SlPR1A* and *SlPR1B* are the marker genes of systemic acquired resistance (SAR). Chitinase (Slcht) can degrade chitin in the cell wall of pathogenic fungi and is considered an important pathogenesis-related protein. The expressions of *SlPR1A* and *SlPR1B* were more strongly induced by *ΔBcdim2rid2* compared to wild-type (Figure 8). Challenging *ΔBcdim2rid2* also significantly promoted the expression of *SlCHT* (Figure 8). Furthermore, we tested the induction effect of *ΔBcdim2rid2* on host resistance by pre-infection. In order to detect the host resistance more quickly and conveniently, we chose tobacco leaves as the host. The results showed that the resistance of the host to the following infection was enhanced by pre-infecting *ΔBcdim2rid2* (Figure S6). These results indicated that *ΔBcdim2rid2* could stimulate stronger disease resistance in the host compared with wild-type and single knockout mutants.

### 3.9. DNA MTases Affect Genomic DNA Methylation of B. cinerea

**Figure 9:**
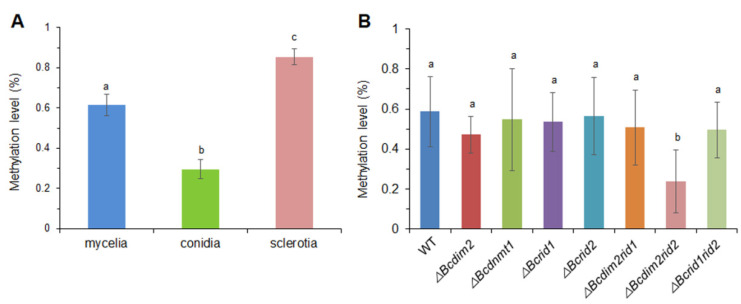
The methylation levels in different tissues and different strains of *B. cinerea*. (**A**) The global methylation levels in different tissues of *B. cinerea*. (**B**) The global methylation levels in wild-type and mutants. A total of 100 ng DNA of each strain was added and fixed on a strip well with a specific affinity for DNA. DNA methylation level was quantified by a 5mC capture antibody and a detection antibody. The amount of methylated DNA was proportional to the optical density (OD) values obtained from the enzyme-linked immunosorbent assays and is presented according to the calculated percentage of 5mC. Columns with different letters indicate significant differences (*p* < 0.05).

We first compared the genomic methylation level in different tissues of wild-type and different strains through immunological methods. The results suggested that the global methylation level of *B. cinerea* was very low, though it possesses more 5mC MTase genes than other species. The conidia harbored the lowest global methylation level (0.3%), and sclerotia had a relatively high methylation level (0.85%) (Figure 9A). Single knockout of *Bcrid1*, *Bcrid2,* and *Bcdnmt1* did not influence the global methylation level; only the single deletion of *Bcdim2* led to the decrease of the methylation level by 20%. The methylation level of *ΔBcdim2rid2* (0.24%) was reduced by 60% compared to the wild-type (0.59%) (Figure 9B).

In order to get the elaborate DNA methylation profile and explore the relationship between methylation pattern and specific gene expression, we then carried out whole genomic high-throughput bisulfite sequencing (BS-Seq) in DNA MTase mutants of *B. cinerea* for the first time, which was deposited in NCBI database (GEO No. GSE131718). Each sample included three biological repeats, and the sequencing depth reached 50×, and the covering rate was higher than 99.99% (Table S3). The sequencing results showed that the global methylation level ranges from 0.28 to 0.44% in all samples, slightly lower than the results measured by immunological methods (Table S4). However, the results of 5mC site detection indicated that few 5mC were detected either in wild-type or MTase mutants after the Binomial Distribution test (Table S5), which preclude us from analyzing the specific regulatory mechanisms of the DNA methylation pattern on the expression of virulence-related genes.

## 4. Discussion

Increasing evidence indicates that pathogenicity of *B. cinerea* to plant hosts can be regulated at multiple levels, including signal transduction [4,5], gene transcription [51,52], and protein secretion [11,12,53]; however, it is still unclear whether an epigenetic modification is involved in the pathogenicity regulation of *B. cinerea*. In this report, we first revealed the important role of DNA methylation in regulating the pathogenicity of *B. cinerea* and explored the functions of 5mC MTases in the development and infection process of *B. cinerea*. DNA methylation appears to be obligatory in plants and mammals but only exists in a subset of fungi, and the methylation level and genotype of 5mC MTases vary among different species [50,54,55]. DNA methylation is widely involved in many biological processes in a lot of organisms, but the biological functions of individual DNA methyltransferases are diverse [56,57,58,59]. In mammals and higher plants, a single DNA methyltransferase can play a vital role in many biological processes such as early embryogenesis, stem cell differentiation, silencing of repetitive elements, X chromosome inactivation, and genomic imprinting [28,29,30,60,61,62,63]. In mice, knockout of *DNMT1*, which is responsible for the maintenance of DNA methylation, resulted in embryonic lethality, with extensive loss of global DNA methylation [64]. The de novo DNA methyltransferases are important for embryogenesis. *DNMT3A/B*-deficient embryos showed growth impairment and multiple developmental defects and eventually died [57]. Defects in DNA methyltransferases in the plant can cause a variety of developmental abnormalities [58,59]. By contrast, the biological function of DNA methyltransferases in fungi seems to be inferior to that in mammals and plants. In *N. crassa*, mutation of *dim-2* led to the elimination of all DNA methylation without causing a detectable phenotype [40]. In this study, we first observed the inhibition effect of DNA methylation inhibitor on pathogenicity and conidial germination of *B. cinerea* (Figure 1A and Figure 2A) and the suppression of the expression of MTase genes during the infection process and conidial germination, indicating that DNA methylation was involved in the pathogenesis and development of *B. cinerea* (Figure 1C and Figure 2E). Genetic analysis showed that knocking out the MTase genes separately did not cause obvious phenotypic variation (Figure 5). Since DIM-2 and RID are two DNA MTases derived from fungi and show high conservation in ascomycetes, we further explore the relationship between them in *B. cinerea*. Most fungi have only one RID protein, while *B. cinerea* has two (BcRID1 and BcRID2), which implies their importance in *B. cinerea*. In *M. robertsii*, MrDIM-2 and MrRID had an additive effect on DNA methylation [42]. In *M. oryzae*, the deletion of *MoRID* resulted in the change of about one-quarter of methylation positions presented in the wild-type, which implied that the knockout of *MoRID* might trigger some compensation mechanism [33]. To better reveal the common mechanism in fungi, we generated three double knockout mutants of the three MTase genes *Bcdim2*, *Bcrid1,* and *Bcrid2* in *B. cinerea* and found that the double knockout mutant *ΔBcdim2rid2* exhibited sharply phenotypic variation. It was almost non-pathogenicity and was impaired in many traits of development (Figure 3, Figure 4 and Figure 5). Particularly, the asexual reproduction of *ΔBcdim2rid2* was dramatically suppressed (Figure 5E), suggesting the important impact of *Bcdim2* and *Bcrid2* on the epidemic of grey mold disease. Unlike the additive effect between *Dim2* and *Rid* in other fungi, *Bcdim2* and *Bcrid2* in *B. cinerea* show a strong complementary effect; namely, the deletion of either one does not cause any phenotypic changes, while the simultaneous absence of both leads to drastic phenotypic changes. This implies a concerted action or functional redundancy between *Bcdim2* and *Bcrid2*; deletion of any one of these two genes can be compensated by the other one, resulting in no obvious functional defect. Our results also suggested that the knockout of *Bcdim2* and *Bcrid2* would raise the expression pattern of *Bcrid2* and *Bcdim2* during conidial germination, respectively (Figure S7) compared with that in the wild-type strain (Figure 2E). However, knocking out two genes simultaneously results in significant phenotypic changes. Among the three double knockout mutants, only *ΔBcdim2rid2* showed obvious phenotypic variation, while the other two had no significant difference compared with the wild-type, which indicated the special interplay mechanism between *Bcdim2* and *Bcrid2*.

To further explore the underlying mechanisms of the virulence impairment of *ΔBcdim2rid2*, we extensively analyzed the expression of genes related to the virulence of *B. cinerea* and the immune response of the host during the interaction progress. Our results suggested that the loss of pathogenicity of double mutant *ΔBcdim2rid2* was attributed to two aspects: the suppression of the expression of pathogenic genes in the mutant and the induction of host resistance by the mutant. The expression patterns of CWDE genes, especially *Bcpg1* and *Bcpg2*, in *ΔBcdim2rid2* were sharply suppressed compared to that in the wild-type (Figure 7). CWDEs have been considered to be the essential weapon of *B. cinerea* for successful infection [2,65], and PGs serve as the important CWDEs employed by *B. cinerea* to facilitate colonization [66,67]. Meanwhile, the expression of other critical virulence factors, including phytotoxin synthesis genes and NADPH oxidase genes, were also significantly suppressed in *ΔBcdim2rid2* (Figure 7). On the other hand, the resistance of the host can be significantly reinforced by *ΔBcdim2rid2* compared to WT and the single knockout mutants. Necrotrophic pathogens usually trigger the JA/ethylene resistant pathway through plant cell surface immune receptors (PRR) [68]. Infection of *ΔBcdim2rid2* led to a stronger expression of several components in JA signaling cascade, including PRR coreceptor (*SlBAK1*), JA pathway marker genes (*SlPI I*), and pathogenesis-related genes (*SlPR1A*/*B*, *SlCHT*), which would greatly enhance the resistance of host (Figure 8). In addition, the expression of the marker gene of SA cascade *SlNPR1* could also be promoted by *ΔBcdim2rid2* relative to WT and the single knockout mutants (Figure 8). The ROS burst in the host plant, mediated by NADPH oxidase RbohD, is one of the most important immune responses to biotrophic pathogens. The local necrosis caused by ROS accumulation can effectively limit the spreading of biotrophic pathogens. However, the cell death of plants caused by ROS is beneficial for the colonization of the typical necrotrophic pathogen *B. cinerea*. The ability of *ΔBcdim2rid2* to induce ROS accumulation in host cells was weaker than that of the wild-type and single knockout mutants, which would inhibit its colonization in host tissues. During the interaction of *ΔBcdim2rid2* and the host, the expression of critical pathogenic factors of *B. cinerea* were inhibited; meanwhile, the ability of *ΔBcdim2rid2* to inhibit the immune response of the host was also obviously weakened, which jointly led to the non-pathogenicity of *ΔBcdim2rid2*.

Through immunological methods, we revealed that *B. cinerea* possessed a low methylation level. Among different tissues of *B. cinerea*, conidia harbored the lowest 5mC level, and sclerotia showed a relatively higher 5mC level (Figure 9A). Unlike other fungi in which DIM-2 is responsible for most of the DNA methylation in the genome, the deletion of *Bcdim2* in *B. cinerea* only resulted in a 20% decrease in global DNA methylation levels. Although the double mutant *ΔBcdim2rid2* was impaired in many traits, its genomic methylation level decreased by only 60%, compared to the wild-type (Figure 9B), suggesting that other MTases also contributed to the global methylation level. Furthermore, we first carried out a whole genome Bs-Seq in different MTase mutants of *B. cinerea* in order to explore the fine regulation mechanism of DNA methylation patterns in this model fungus. However, low methylation levels were detected in all strains, which were comparable to the non-conversion rate of Bs-Seq (Table S4). In general, the genotype of 5mC DNA MTases is the top predictor of genomic CG methylation level. Although *B. cinerea* possesses multiple 5mC MTases, the genomic DNA methylation level is very low. By contrast, *Pseudogymnoascus destructans*, which possesses the same genotype of MTase as *B. cinerea*, has a high DNA methylation level [50]. This seemingly contradictory phenomenon may be attributed to the following reasons. First, the tissue-specific expression divergence of MTase genes between *P. destructans* and *B. cinerea* might be an explanation. In *B. cinerea*, MTase genes had higher expression in sexual reproduction structure but lower expression in mycelia, which was used for the determination of the DNA methylation level of mutants in this study [50]. In contrast, all the 5mC MTases showed a relatively higher expression level in the mycelia of *P. destructans* [50]. Second, 5mC is mutagenic and can cause spontaneous deamination of methylated cytosine to thymine (T), which was often triggered by RIP, resulting in the depletion of 5mC over evolutionary progress. In filamentous fungi, RIP occurs frequently and led to multiple C to T transition mutations in repeated sequences, which is considered a defense against the spread of transposable elements [69]. Therefore, the methylated cytosine may be a transient intermediate state during this process, and the method of Bs-Seq is unable to detect the methylation state in this process. DNA MTase RID is responsible for the RIP progress. The expansion of the RID number and the high AT content may partially explain the lower methylation level of 5mC in *B. cinerea*. In addition, since bisulfite treatment has a non-conversion rate (about 0.3–0.5%), it is difficult to accurately detect the methylation level close to the non-conversion rate. Therefore, monitoring the DNA methylation status in fungi with extremely low methylation levels may need to develop more accurate or targeted methods in the future.

In brief, we unravel the important function of DNA MTases in *B. cinerea*. We found the synergistic effects of two MTases, BcDIM2 and BcRID2, in the development and infection process of *B. cinerea.* Double knockout of *Bcdim2* and *Bcrid2* significantly altered the interaction between *B. cinerea* and horticultural hosts. These findings provide a new perspective into the regulation mechanism of the pathogenicity of *B. cinerea* to horticultural crops at the epigenetic level, which is beneficial for understanding the complex infection mechanism and elaborate regulatory network of the fungal pathogen during infection of horticultural crops.

## Data Availability

The methylome data of different strains were deposited in GEO. The GEO accession number is “GSE131718”.

## Supplementary Materials

The following are available online at https://www.mdpi.com/article/10.3390/jof7080659/s1, Figure S1: Analysis of DNA methyltransferases of B. cinerea. Figure S2: Knockout strategy of target genes. Figure S3: PCR diagnosis of knockout mutants. Figure S4: Southern blot analysis of DNA MTase mutants. Figure S5: Activities of extracellular pathogenic proteins are down-regulated in double knockout mutant ΔBcdim2rid2. Figure S6: Pre-infection of ΔBcdim2rid2 induced the resistance of host. Figure S7: The expression pattern of Bcdim2 in ΔBcrid2 (A) and the expression pattern of Bcrid2 in ΔBcdim2 during conidial germination. Table S1: The primers used for generation of knockout mutants. Table S2: The primer sequences for RT-qPCR. Table S3: The statistics of whole-genome bisulfite sequencing for 12 samples. Table S4: Global DNA methylation levels of different strains. Table S5: The numbers of methylated cytosines in each sample.
