# Supplementary material for: DNA Methyltransferases Regulate Pathogenicity of Botrytis cinerea to Horticultural Crops
Source: J Fungi (Basel). 2021 Aug 14;7(8):659. doi: 10.3390/jof7080659 (PMC8399656; doi:10.3390/jof7080659)
Supplement: Supplementary file 1 [file jof-07-00659-s001.zip › jof-1320962-SI.pdf]

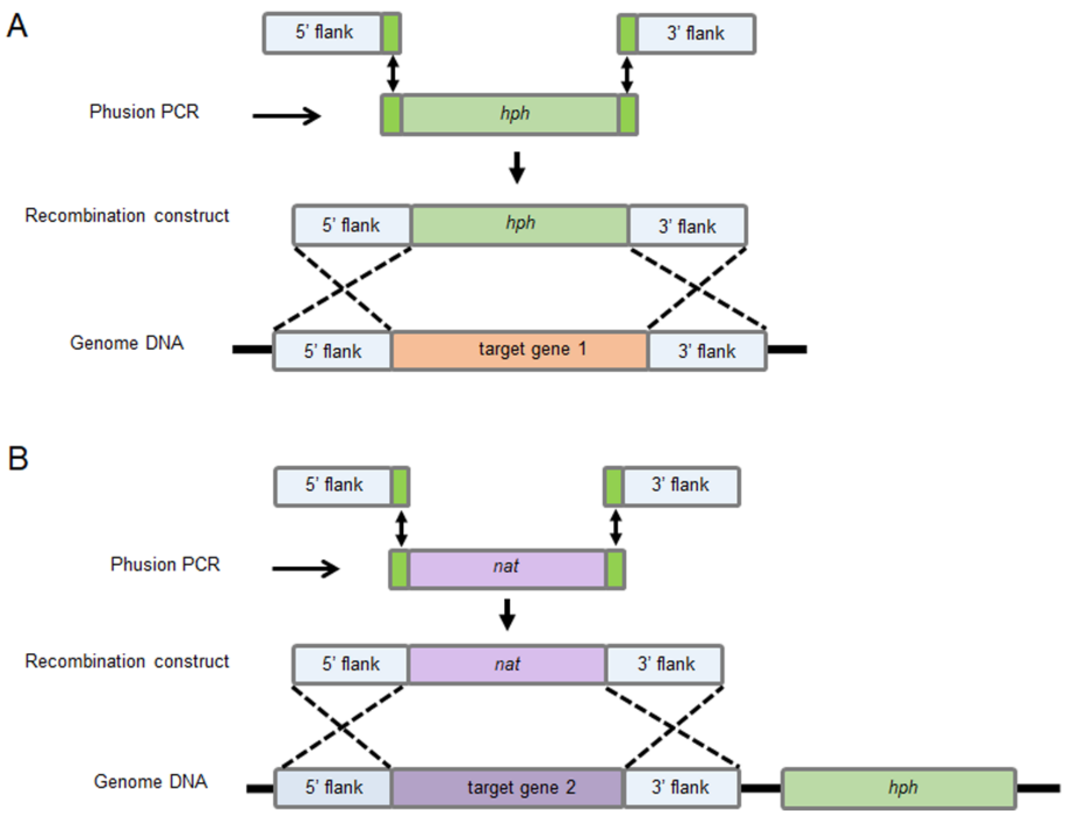


**Figure S1** Knockout strategy of target genes. (A**)** Strategy for single knockout. *hph*: hygromycin-resistance cassette. (B)Strategy for double knockout. *nat*: nourseothricin -resistance cassette.

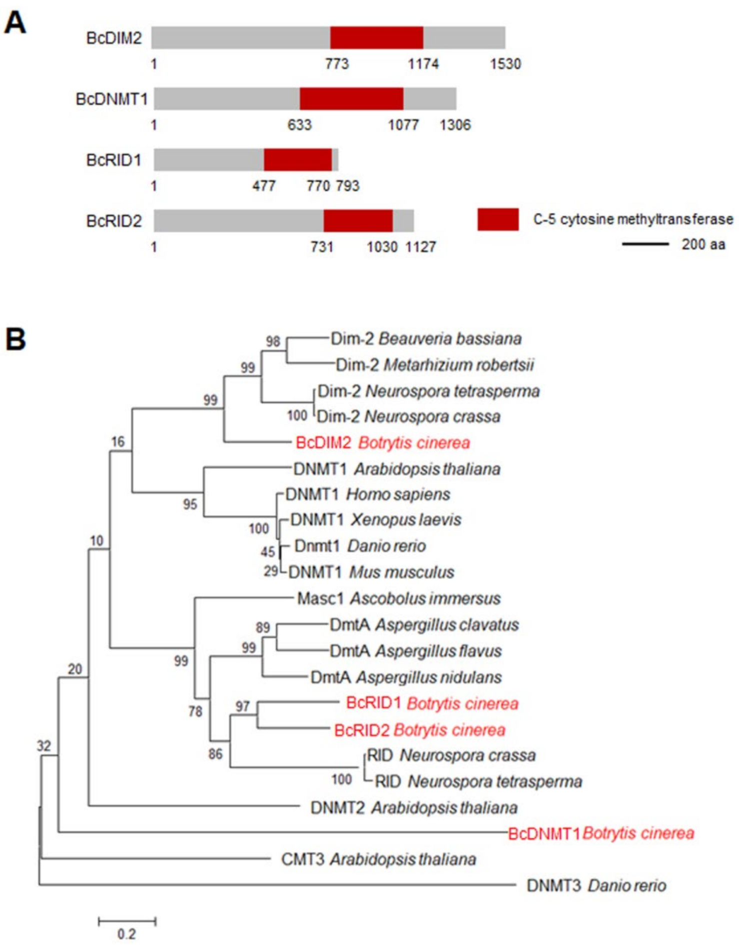


**Figure S2** Analysis of DNA methyltransferases of *B. cinerea*. (A) Schematic diagram of conserved domain of C5 cytosine methyltransferase. (B) Phylogenetic tree of the of 5mC MTase proteins in *B. cinerea*.

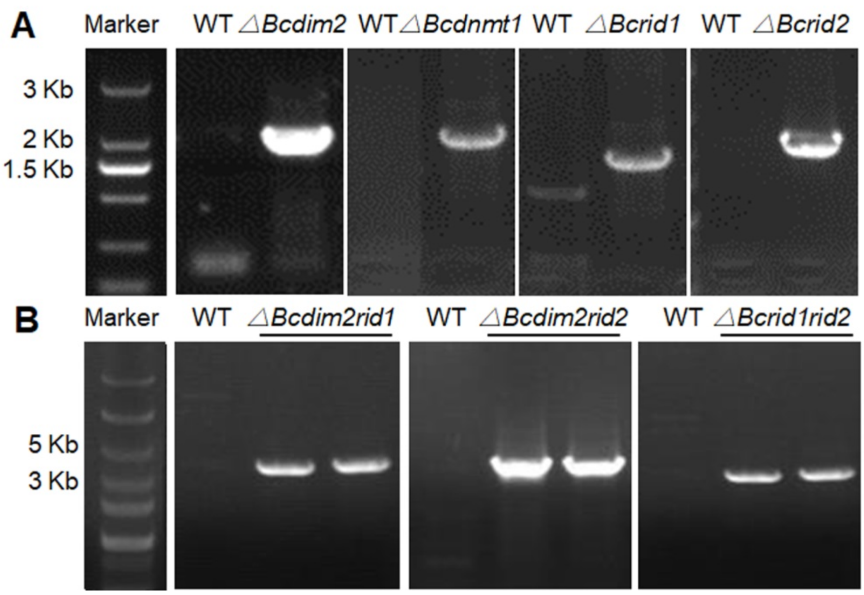


**Figure S3** PCR diagnosis of knockout mutants. Flank-spanning PCR for single knockout mutants (A) and double knockout mutants (B).

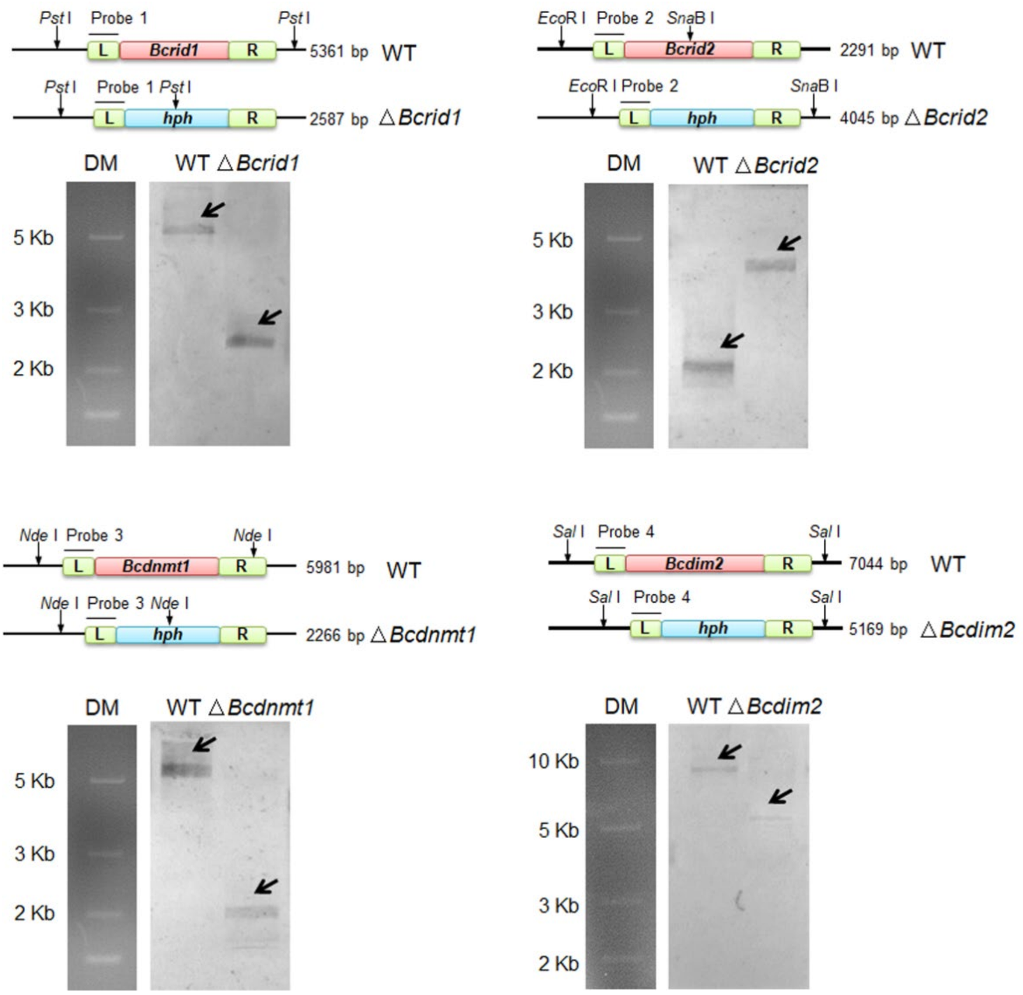


**Figure S4** Southern blot analysis of DNA MTase mutants.

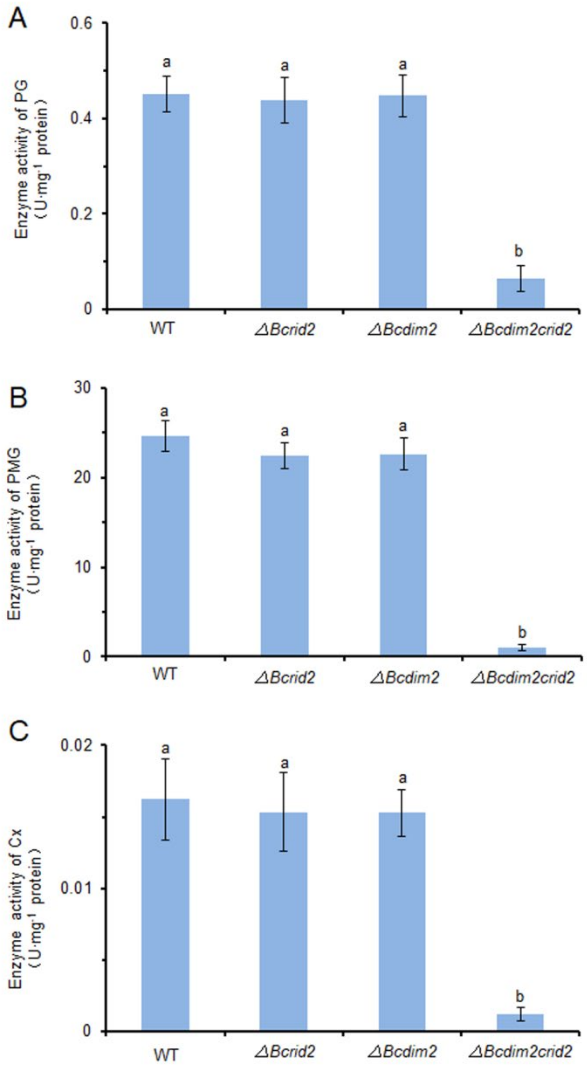


**Figure S5** Activities of extracellular pathogenic proteins are down-regulated in double knockout mutant △*Bcdim2rid2*. (A) Activity of polygalacturonase (PG). (B) Activity of poly-methyl galacturonate (PMG). (C) Activity of carboxymethyl cellulose (Cx).

A

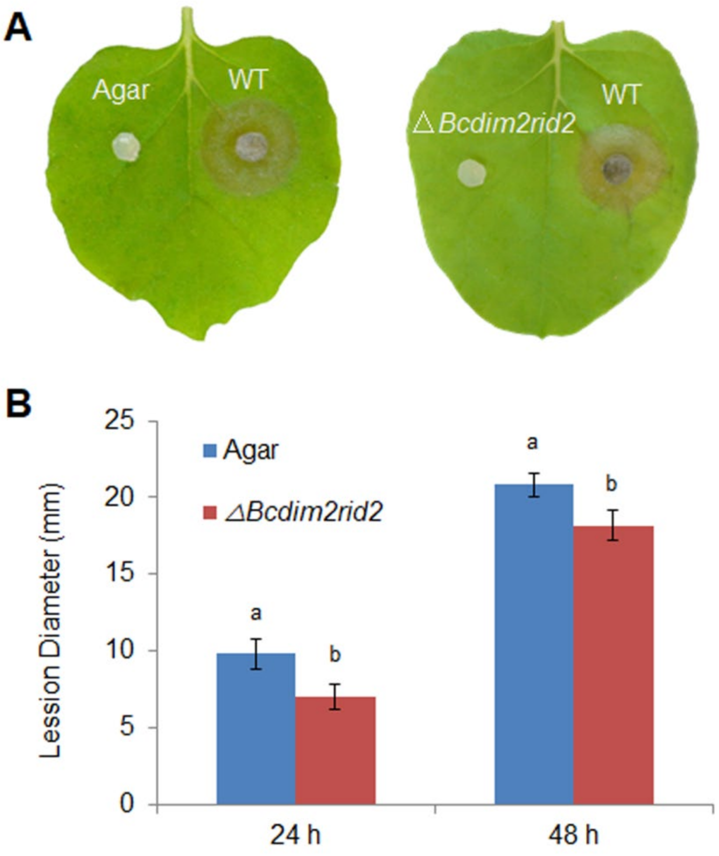


**Figure S6** Pre-infection of *△Bcdim2rid2* induced the resistance of host. (A) Disease symptoms on detached tobacco leaves (48 hpi). (B) Lesion diameters on detached tobacco leaves.

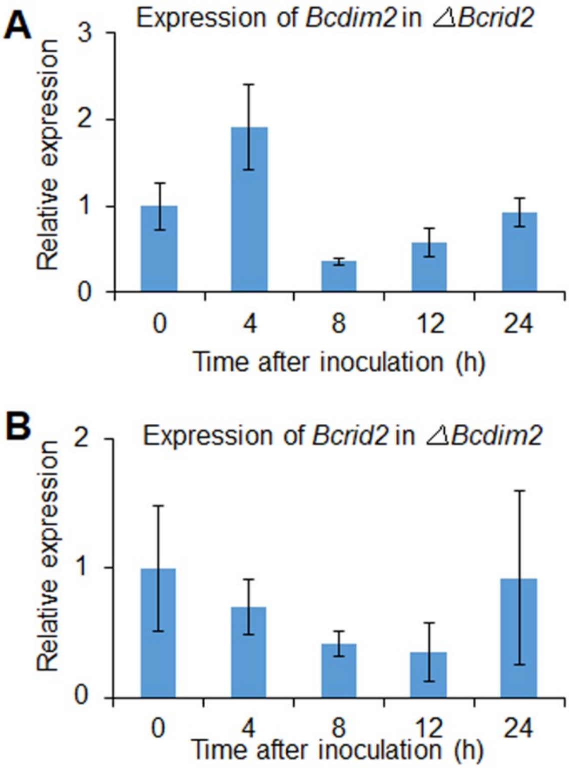


**Figure S7** The expression pattern of *Bcdim2* in △*Bcrid2* (A) and the expression pattern of *Bcrid2* in △*Bcdim2* during conidial germination.

**Table S1** The primers used for generation of knockout mutants.

| **Primers name** | **Sequence (5'→3')** |
|---|---|
| Bcdim2-uF | CTCCACCAACTGCAATCACT |
| Bcdim2-uR | AATCGGAATGCGGCTCCACAATAAAGCCGAGCATACGAAA |
| Bcdim2-mF | TTTCGTATGCTCGGCTTTATTGTGGAGCCGCATTCCGATT |
| Bcdim2-mR | CTCTTCCCCTTTCTCTCCAAAGGCAACCATGCATGGTTAC |
| Bcdim2-dF | GTAACCATGCATGGTTGCCTTTGGAGAGAAAGGGGAAGAG |
| Bcdim2-dR | ATTAGTTTGGCGAGTGGATG |
| Bcrdnmt1-uF | CGATAACAAACCTCGGAATT |
| Bcdnmt1-uR | AATCGGAATGCGGCTCCACAATCTACGCAAACTTGTCTCA |
| Bcdnmt1-mF | TGAGACAAGTTTGCGTAGATTGTGGAGCCGCATTCCGATT |
| Bcdnmt1-mR | CACATAGACGCTTGTCAATCAGGCAACCATGCATGGTTAC |
| Bcdnmt1-dF | GTAACCATGCATGGTTGCCTGATTGACAAGCGTCTATGTG |
| Bcdnmt1-dR | GAGCCAAGTGAGAAAGAACG |
| Bcrid1-uF | CTACCTTCTGCTCGGCTTCT |
| Bcrid1-uR | AATCGGAATGCGGCTCCACACCCTTACTGCCCTCTCTCTT |
| Bcrid1-mF | AAGAGAGAGGGCAGTAAGGGTGTGGAGCCGCATTCCGATT |
| Bcrid1-mR | TGTTTTCTGTATGCCAGTTTAGGCAACCATGCATGGTTAC |
| Bcrid1-dF | GTAACCATGCATGGTTGCCTAAACTGGCATACAGAAAACA |
| Bcrid1-dR | ATACCACAGAGGCTGAACAA |
| Bcrid2-uF | CAATCTTCCACTCCCTCATC |
| Bcrid2-uR | AATCGGAATGCGGCTCCACACAGTAGGTAGCAGGTCAAAC |
| Bcrid2-mF | GTTTGACCTGCTACCTACTGTGTGGAGCCGCATTCCGATT |
| Bcrid2-mR | CCATTCCCACTTCCACTTTTAGGCAACCATGCATGGTTAC |
| Bcrid2-dF | GTAACCATGCATGGTTGCCTAAAAGTGGAAGTGGGAATGG |
| Bcrid2-dR | GTGAGTTAGTGGCTCGGATA |
| Bcdim2-uR2 | ATGCGGCTCTAGAGGATCCCATAAAGCCGAGCATACGAAA |
| Bcdim2-mF2 | TTTCGTATGCTCGGCTTTATGGGATCCTCTAGAGCCGCAT |
| Bcdim2-mR2 | CTCTTCCCCTTTCTCTCCAACGCCGAGTTAGGCAACCATG |
| Bcdim2-dF2 | CATGGTTGCCTAACTCGGCGTTGGAGAGAAAGGGGAAGAG |
| Bcrid1-uR2 | ATGCGGCTCTAGAGGATCCCCCCTTACTGCCCTCTCTCTT |
| Bcrid1-mF2 | AAGAGAGAGGGCAGTAAGGGGGGATCCTCTAGAGCCGCAT |
| Bcrid1-mR2 | TGTTTTCTGTATGCCAGTTTCGCCGAGTTAGGCAACCATG |
| Bcrid1-dF2 | CATGGTTGCCTAACTCGGCGAAACTGGCATACAGAAAACA |
| Bcrid2-uR2 | ATGCGGCTCTAGAGGATCCCCAGTAGGTAGCAGGTCAAAC |
| Bcrid2-mF2 | GTTTGACCTGCTACCTACTGTGTGGAGCCGCATTCCGATT |
| Bcrid2-mR2 | CCATTCCCACTTCCACTTTTAGGCAACCATGCATGGTTAC |
| Bcrid2-dF2 | GTAACCATGCATGGTTGCCTAAAAGTGGAAGTGGGAATGG |
| Bcdnmt5-uF | ATTGATAGGTGGACGAGACT |
| Bcdnmt5-uR | AATCGGAATGCGGCTCCACAGAGATGGAATGATTGAGAAA |
| Bcdnmt5-mF | TTTCTCAATCATTCCATCTCTGTGGAGCCGCATTCCGATT |
| Bcdnmt5-mR | ACAACATGAGCCTAACAGCTAGGCAACCATGCATGGTTAC |

| | |
|---|---|
| Bcdnmt5-dF | GTAACCATGCATGGTTGCCTAGCTGTTAGGCTCATGTTGT |
| Bcdnmt5-dR | CATATTTGAGTTCGCTGGAT |
| Bcdim2-detect-F | ACTGTATGGCCCCCTCTACC |
| Bcdnmt1-detect-F | CAAAGGTTATGAGGAAGGCC |
| Bcrid1-detect-F | TTGACAGCCAATTCCATACC |
| Bcrid2-detect-F | TTCCTCGTTCCTTGGGTGCT |
| Bcdnmt5-detect-F | TTTGCCGTTCGTGTTATTGT |
| hph-detect-R | GTTTGCTGGTTACGCTTT |
| nat-detect-R | ATTCTATCCCTGGCTTTCCC |

**Table S2** The primer sequences for RT-qPCR.

| Gene names | Primer names | Sequences （5‘-3’ ） |
|---|---|---|
| *Bcpg1* | Bcpg1-q-F | GATGTTGGTTCCTCCAGCGATA |
| | Bcpg1-q-R | CCGGAGTTGATAGCGAGACAGT |
| *Bcpg2* | Bcpg2-q-F | CGCCCCAGCAGATCTTGATA |
| | Bcpg2-q-R | AGCAGGTGGTCTTTGAGGCTAT |
| *Bcxyn11a* | Bcxyn11a-q-F | ACATACTCCAACGGAGCCAATG |
| | Bcxyn11a-q-R | CATCCTTTTCCACCGACGAA |
| *BcnoxA* | BcnoxA-q-F | TCGCGTCTCCGTTGTATGAA |
| | BcnoxA-q-R | TCCCTGTGGCTTGATTAACCA |
| *BcnoxB* | BcnoxB-q-F | GGTCCAAAAGTGTTGGGTAGTCA |
| | BcnoxB-q-R | TTGCCCCAGCAGAAATTGA |
| *BcnoxD* | BcnoxD-q-F | AGTGATAGCAGGTGTTTGGAAGCT |
| | BcnoxD-q-R | AGCACGCCATCATGTCCTTT |
| *BcnoxR* | BcnoxR-q-F | ACCCAATTCGTTCTGCTTCGT |
| | BcnoxR-q-R | ACCGAATGAAGATGCGGATAA |
| *Bcrho3* | Bcrho3-q-F | CTTCTTCCTCCTGCCAACCA |
| | Bcrho3-q-R | TGATTGCGAAGGAATGCCTAT |
| *Bcsod1* | Bcsod1-q-F | ATCTCCGGCACCGTCACTT |
| | Bcsod1-q-F | ATCTCCGGCACCGTCACTT |
| *Bcbmp1* | Bcbmp1-q-F | TCTTTCAATGTCAGCGAGCAA |
| | Bcbmp1-q-R | TGCAAAGCTGAGCAGACAACA |
| *BcG1* | BcG1-q-F | ACCACGGGTATCACCGAAAC |
| | BcG1-q-R | TCGGAACGTTGTCCTCCAA |
| *Bcbtp1* | Bcbtp1-q-F | GGCCGTGTTGTGATCGAAGT |
| | Bcbtp1-q-R | AGCTTTTCAGGGCGGATGT |
| *Bcplc1* | Bcplc1-q-F | TCCCGCAGGACTCGATAACT |
| | Bcplc1-q-R | TATGGCTTCCACTCGGGTTT |
| *Bcbos5* | Bcbos5-q-F | ACCCACTTCCATGGCAGAAA |
| | Bcbos5-q-R | CGGGTAACTGTGGTCGTTGTG |
| *Bcbac* | Bcbac-q-F | CCGTGTCGCTTACCAAGAAAC |
| | Bcbac-q-R | TTGCATCGCTGGTAGACCATT |
| *Bcras2* | Bcras2-q-F | TCTTCCCTCGACAGCGTTTG |
| | Bcras2-q-R | ACGCCGTATGCCACTCTTTT |
| *Bcmsb2* | Bcmsb2-q-F | CTCACTCCGACCTCACCTCTTC |
| | Bcmsb2-q-R | TTCGCTCTCGAGTTCGATTGT |
| *Bccrz1* | Bccrz1-q-F | CATGCAACAGCAGCCTATGG |
| | Bccrz1-q-R | GTGCAGCAGGTAAGCCGTAAT |
| *Bcste12* | Bcste12-q-F | CCCCACTCGATTTAGTTGACATC |
| | Bcste12-q-R | TCATATGCGCCATTCAATTGAG |
| *Bchox8* | Bchox8-q-F | ATGTCAATGATGCACCGAAGTG |
| | Bchox8-q-R | CCGGTACCATCATCGGAGAT |
| *Bcmads1* | Bcmads1-q-F | GAAGGCAAAGCTCCCTGGTA |

| | | |
|---|---|---|
| | Bcmads1-q-R | TGATGGATTTGGCGCATACA |
| *Bcboa2* | Bcboa2-q-F | GGCTTGTCGAGACTACCGATCT |
| | Bcboa2-q-R | TGCTCCTAAAGCGCCGATAA |
| *Bcboa6* | Bcboa6-q-F | CTCTTACGCTTACATGCGTCACTT |
| | Bcboa6-q-R | CCAGTTAAACAAAGGACGGTGAT |

**Table S3.** The statistics of whole-genome bisulfite sequencing for 12 samples

| Samples | Clean data | Mapping rate | Conversion rate | Coverage | Coverage (d>=5) |
|---|---|---|---|---|---|
| **WT-1** | 2.2 G bp | 63.4% | 99.6% | 99.99% | 94.4% |
| **WT-2** | 2.1 G bp | 61.8% | 99.5% | 99.99% | 94.0% |
| **WT-3** | 1.9 G bp | 62.3% | 99.6% | 99.99% | 92.3% |
| ***△Bcdim2*-1** | 2.1 G bp | 76.6% | 99.6% | 99.99% | 97.3% |
| ***△Bcdim2*-2** | 2.5 G bp | 77.0% | 99.6% | 99.99% | 98.5% |
| ***△Bcdim2*-3** | 2.1 G bp | 78.5% | 99.6% | 99.99% | 97.8% |
| ***△Bcrid2*-1** | 2.2 G bp | 64.1% | 99.6% | 99.99% | 96.1% |
| ***△Bcrid2*-2** | 2.3 G bp | 66.3% | 99.6% | 99.99% | 96.6% |
| ***△Bcrid2*-3** | 2.2 G bp | 86.5% | 99.6% | 99.99% | 98.5% |
| ***△Bcdim2rid2*-1** | 2.3 G bp | 86.2% | 99.7% | 99.99% | 98.8% |
| ***△Bcdim2rid2*-2** | 2.0 G bp | 87.0% | 99.6% | 99.99% | 96.6% |
| ***△Bcdim2rid2*-3** | 2.0 G bp | 85.9% | 99.6% | 99.99% | 96.2% |

**Table S4.** Global DNA methylation levels of different strains

| | **C** | | | **CG** | | | **CHG** | | | **CHH** | | |
|---|---|---|---|---|---|---|---|---|---|---|---|---|
| | Repeat1 | Repeat2 | Repeat3 | Repeat1 | Repeat2 | Repeat3 | Repeat1 | Repeat2 | Repeat3 | Repeat1 | Repeat2 | Repeat3 |
| **WT** | 0.38% | 0.44% | 0.36% | 0.34% | 0.40% | 0.32% | 0.37% | 0.43% | 0.36% | 0.39% | 0.45% | 0.37% |
| ***△Bcdim2*** | 0.37% | 0.39% | 0.43% | 0.33% | 0.36% | 0.39% | 0.37% | 0.40% | 0.44% | 0.38% | 0.40% | 0.44% |
| ***△Bcrid2*** | 0.39% | 0.39% | 0.43% | 0.36% | 0.36% | 0.39% | 0.39% | 0.40% | 0.43% | 0.40% | 0.40% | 0.44% |
| ***△Bcdim2rid2*** | 0.28% | 0.43% | 0.37% | 0.25% | 0.39% | 0.34% | 0.28% | 0.43% | 0.37% | 0.29% | 0.44% | 0.39% |

**Table S5** The numbers of methylated cytosines in each sample

| Samples | Total C | mC | mC% |
|---|---|---|---|
| **WT-1** | 16851479 | 37 | 0.0002% |
| **WT-2** | 16779126 | 35 | 0.0002% |
| **WT-3** | 16486434 | 42 | 0.0002% |
| △***Bcdim2-1*** | 17406799 | 34 | 0.0002% |
| △***Bcdim2-2*** | 17625101 | 33 | 0.0002% |
| △***Bcdim2-3*** | 17487257 | 38 | 0.0002% |
| △***Bcrid2-1*** | 17178586 | 32 | 0.0002% |
| △***Bcrid2-2*** | 17278470 | 45 | 0.0002% |
| △***Bcrid2-3*** | 17620206 | 76 | 0.0002% |
| △***Bcdim2rid2-1*** | 17667930 | 13 | 0.0002% |
| △***Bcdim2rid2-2*** | 17255226 | 91 | 0.0002% |
| △***Bcdim2rid2-3*** | 17196563 | 73 | 0.0002% |
